# Targeted Lipid Nanoparticles Encapsulating Dihydroartemisinin and Chloroquine Phosphate for Suppressing the Proliferation and Liver Metastasis of Colorectal Cancer

**DOI:** 10.3389/fphar.2021.720777

**Published:** 2021-10-08

**Authors:** Jianqing Peng, Qin Wang, Jia Zhou, Shuli Zhao, Pan Di, Yan Chen, Ling Tao, Qianming Du, Xiangchun Shen, Yi Chen

**Affiliations:** ^1^ State Key Laboratory of Functions and Applications of Medicinal Plants, Guizhou Medical University, Guiyang, China; ^2^ High Efficacy Application of Natural Medicinal Resources Engineering Center of Guizhou Province, School of Pharmaceutical Sciences, Guizhou Medical University, Guiyang, China; ^3^ General Clinical Research Center, Nanjing First Hospital, Nanjing Medical University, Nanjing, China; ^4^ Department of Clinical Pharmacy, School of Basic Medicine and Clinical Pharmacy, China Pharmaceutical University, Nanjing, China

**Keywords:** chloroquine phosphate, lipid nanoparticles, colorectal cancer, liver metastasis, ROS, dihydroartemisinin

## Abstract

Antimalarial drugs Dihydroartemisinin (DHA) and chloroquine phosphate (CQ) exhibit evident anti-cancer activity, particularly as combination therapy. DHA and CQ combination therapy has been proved to exhibit higher cytotoxic effect in tumor cells and lower toxicity to normal cells than combination of artemisinin derivatives (ARTs) and anticancer chemotherapy drugs. However, different physiochemical properties of DHA and CQ, leading to distinctive *in vivo* outcomes, considerably limited their synergistic effect in cancer treatment. Herein, we developed a lipid nanoparticle (LNP) for co-delivery of DHA and CQ to inhibit proliferation and metastasis of colorectal cancer. Considering the beneficial effects of acid/reactive oxide species (ROS)-sensitive phospholipids and targeting ligands for colorectal cancer cells, an RGD peptide-modified pH/ROS dual-sensitive LNP loaded with DHA and CQ (RLNP/DC) was prepared. It exhibited optimal cytotoxicity and suppression of invasion and metastasis in HCT116 cells *in vitro*, attributable to irreversible upregulation of intracellular ROS levels, downregulation of VEGF expression, and upregulation of paxillin expression. A mouse model of orthotopic metastasis of colorectal cancer was established to evaluate anti-proliferation and anti-metastasis effects of RLNP/DC *in vivo*. Thus, an optimized nanoplatform for DHA and CQ combination therapy was developed in this study that offered potential antitumor efficacy against colorectal cancer.

## Introduction

Colorectal cancer is the third most common cancer diagnosed and the second leading cause of cancer-related deaths worldwide in 2020 (Sung et al., 2021). Both orthotopic proliferation and remote metastasis lead to progression and recurrence of colorectal cancer, which is the main cause of treatment failure and mortality. Chemotherapy is one of the major treatment strategies for stage II–IV colorectal cancer according to the clinical practice guidelines, which recommends combination therapies owing to their synergistic effects. However, combination of small drug molecules or monoclonal antibodies generally exhibits side effects to some extent because of their high toxicity to normal tissues. Therefore, combination therapy with drugs having selective anti-proliferation and anti-metastasis effects on tumor cells is promising for the treatment of colorectal cancer.

Dihydroartemisinin (DHA) is a derivative of artemisinin (ART), which has been reported to exhibit broad-spectrum anticancer activity (Slezáková and Ruda-Kucerova, 2017; Wong et al., 2017). It induces cell cycle arrest, apoptosis, and ferroptosis of tumor cells mainly through generation of reactive oxygen species (ROS). Although ART-based drugs showed excellent clinical safety with low incidence of side effects, they are not recommended to be used alone for cancer treatment due to their low potency. ART-based drugs elevate intracellular ROS levels via cleavage of the endoperoxide bridge, which causes mitochondrial dysfunction and caspase 3-dependent or Bax-mediated apoptosis in various tumor cells (Bhaw-Luximon and Jhurry, 2017). However, autophagy plays a key role in ROS-dependent tumor regression, which is partially restricted by the intracellular antioxidant system. Paradoxically, autophagy has been observed in cancer cells. It contributes to the intracellular recycling of ferrous iron (Fe^2+^), which mediates ROS generation by DHA as well as ferroptosis (Hou et al., 2016). In addition, it degrades damaged organelles and components leading to ROS hemostasis, thereby reducing DNA damage, which prevents oxidative stress against cell death (Lee et al., 2012). Thus, autophagy is a potential factor that determines anticancer effect of DHA.

Chloroquine phosphate (CQ), an effective autophagy inhibitor, has been reported to prevent the formation of autolysosomes by increasing the pH of lysosomes via protonation (Redmann et al., 2017). It has been shown to improve the therapeutic effects of anticancer drugs, particularly autophagy-inducing drugs (Torgersen et al., 2013). ART and its analogs have been used in cancer treatment along with autophagy inhibitors and their combination have shown to exert synergistic anticancer activity (Button et al., 2014; Ganguli et al., 2014). Considering ROS regulation mechanisms of DHA and CQ, the combination was proposed to exhibit selective cytotoxicity in colorectal cancer cells. High concentration of Fe^2+^ in colorectal cancer cells mediates DHA-induced ROS generation; the intracellular ROS level is further amplified by CQ by reducing ROS elimination. In contrast, low basal level of Fe^2+^ in colonic epithelial cells attenuates DHA-induced ROS generation, and CQ-induced autophagy inhibition might result in inconsiderable elevation in ROS levels. Therefore, we propose that DHA and CQ might be a promising combination therapy against colorectal cancer exhibiting anti-proliferation effect through irreversible ROS upregulation via facilitation of ROS generation and suppression of autophagy.

Moreover, DHA and CQ have been reported to prevent the metastatic spread of tumor cells to remote organs via different mechanisms. Notably, tumor cells at the primary lesion need to overcome the extracellular matrix (ECM) adhesion, and then freely transport through tumor-associated stoma to systemic circulation. CQ has been reported to reduce the degradation of paxillin, which supports anchoring of tumor cells to the primary tumor lesion, and prevents the first crucial step of metastasis (Wang et al., 2018). Inhibition of autophagy-dependent disassembly of focal adhesions maintains the attachment between paxillin in tumor cells and integrin on the ECM. Although the mechanism underlying autophagy as a regulator of metastasis is not entirely clear, it helps cells to adapt to stressful tumor microenvironment. It is reasonable to propose that CQ, an autophagy inhibitor, has great potential to reduce migration and invasion of colorectal cancer cells mediated by distant metastases of the primary tumor. In addition, tumor vascularization is necessary for distant metastases of the primary tumor after detachment from the ECM. ART-based drugs have been shown to inhibit angiogenesis in several malignancies by suppressing the secretion of angiogenic factors, such as vascular endothelial growth factor (VEGF) and angiopoietin 1 (Ang-1) (Chen et al., 2010; Saeed et al., 2015). Thus, DHA and CQ might synergize to prevent metastasis of colorectal cancer owing to the antiangiogenic activity and maintain the focal adhesion of tumor cells to the ECM. Collectively, the combination of DHA and CQ has great potential for suppressing the proliferation and metastasis of colorectal cancer.

Nevertheless, different physicochemical properties of DHA and CQ, non-selectivity, and unsynchronized biodistribution to the tumor region limited their inhibitory effects against colorectal cancer *in vivo*. Lipid nanoparticles (LNPs) with a repository for both hydrophilic and lipophilic drugs are ideal nanocarriers, which might prolong the circulation time and achieve synchronized biodistribution of DHA and CQ to the tumor region. Considering the importance of rapid and complete drug release from LNPs for exploiting synergistic effects of combination therapies, overcoming intracellular obstacles and delivering cargos after complete destruction of these particles are the rate-limiting steps of the treatment. 1,2-dioleoyl-sn-glycero-3-phosphoethanolamine (DOPE)-based liposomes have fusogenic potential to escape from lysosomes via phase transition of DOPE in an acidic environment (Simões et al., 2004). Soybean phospholipids (SPC), DOPE, and 1,2-dioleoyl-sn-glycero-3-phosphocholine (DOPC), each containing carbon–carbon double bonds (C=C), are sensitive to elevated ROS levels (Ogilby, 2010). The unsaturated fatty acids in phospholipids are liable to be oxidized to lipid peroxides, resulting in enhanced hydrophilicity and disrupted integrity of the lipid layer. Therefore, LNPs composed of SPC, DOPE, and DOPC are likely to be pH/ROS-responsive, leading to intracellular disruption and drug release. Intriguingly, irreversible ROS upregulation by DHA and CQ combination, which resulted in self-amplified ROS signaling, could further promote the disruption of LNPs and release of cargos.

In this study, we aimed to develop pH/ROS dual-responsive LNPs for combination therapy of DHA and CQ (LNP/DC) to suppress proliferation and liver metastasis of colorectal cancer. The lipid layer of LNPs was composed of SPC, DOPE, and DOPC. DHA and CQ were encapsulated *via* consecutive drug loading process using the film dispersion method for DHA and the remote loading method for CQ. A cyclic arginylglycylaspartic acid (RGD) peptide as a specific ligand of α_v_β_3_ integrin overexpressed in colorectal cancer and neovascular cells, has been widely used in nanodevices (Biscaglia et al., 2019; Peng et al., 2019). RGD was coated on the surface of LNPs *via* DSPE-PEG-RGD to form RLNP/DC, endowing it with colorectal cancer targeting capability. The findings of the present study confirmed that RLNP/DC considerably enhanced the anti-proliferative and anti-metastatic effects of combination therapy with DHA and CQ in colorectal cancer, attributable to the targeted and synchronized delivery to the primary tumor region, pH/ROS-responsive release of DHA and CQ, and ingeniously formed self-amplified intracellular ROS signaling.

## Materials and Methods

### Materials

SPC (purity ≥ 98%, lot no. SY-SI-190601) was obtained from Lipoid (Ludwigshafen am Rhein, Germany). Cholesteryl oleate (CO, purity ≥ 85%), glycerol trioleate (GT, purity ≥ 97%, lot no. D1915109), DHA (purity ≥ 98%, lot no. J1818126), and sodium 1-hexanesulfonate (purity ≥ 98%) were obtained from Aladdin Reagent Co., Ltd. (Shanghai, China). CQ (purity ≥ 98%, lot no. C10331390) and cyanine-5.5 (purity ≥ 95%, lot no. C11249895) was purchased from Macklin Biochemical Co., Ltd. (Shanghai, China). Calcein (lot no. LJC0R33) was obtained from J&K Chemical Co., Ltd. (Beijing, China). DOPE, DOPC, 1,2-distearoyl-sn-glycero-3-phosphoethanolamine-N-[methoxy(polyethylene glycol)-2000] (DSPE-PEG_2000_, lot no. RJ0190413), and DSPE-PEG_2000_-cRGD (lot no. RJ0190813) were provided by Xi’an ruixi Biological Technology Co., Ltd. (Shaanxi, China). Chromatographic grade organic reagents were obtained from Aladdin Reagent Co., Ltd.

### Cell Culture

Human colorectal cancer cell line HCT116 and SW480 was obtained from the American Tissue Culture Collection (ATCC, Rockville, MD, United States), and human colonic epithelial cell line NCM460 was obtained from Incell (San Antonio, TX, United States). Both cell lines were validated by short tandem repeat DNA fingerprinting at Guangzhou Cellcook Biotech Co., Ltd. (Guangdong, China). HCT116 cells, SW480 cells, and NCM460 cells were respectively cultured in McCoy’s 5A medium (Gibco, Invitrogen Corporation, NY, United States), Leibovitz’s L-15 medium (Gibco, Invitrogen Corporation, NY, United States) and RPMI 1640 medium (Gibco, Invitrogen Corporation, NY, United States) supplemented with 10% fetal bovine serum (FBS, Hyclone, Logan, UT, United States). In the following experiments, both cell lines were used within 10 passages.

### Preparation of DHA/CQ-Loaded LNPs

DHA and CQ co-encapsulated LNPs were prepared using a sequential drug loading process. DHA was loaded using the thin lipid film dispersion method; CQ was subsequently encapsulated using the remote loading method. Briefly, SPC, DOPE, DOPC, CO, GT, and DHA were dissolved in chloroform at a mass ratio of 10:5:5:5:5:1. DSPE-PEG_2000_ dissolved in ethanol was added to total lipids at a mass ratio of 1:20. After vortexing for 10 min, rotary evaporator (RE-52AA, Beidi Experimental Instrument, Nanjing, China) was used to remove the organic solvent and obtain a thin lipid film. Then, 4 ml of ammonium sulfate solution (0.2 M) for hydration was added in a water bath at 60°C for 1 h. The hydration solution was sonicated at 260 W for 5 min including 3 s pulses, separated by 3 s pauses, and then passed through a 0.45 μm filter. Thus obtained LNP/DHA was dialyzed (molecular weight cut-off, MWCO 14 kDa) against 0.9% sodium chloride for 3 h. Afterwards, LNP/DHA was incubated with CQ solution (20 mg/ml) at a total lipids/CQ mass ratio of 80:3 in a water bath at 50°C for 30 min. The free CQ was removed via dialysis against phosphate buffered saline (PBS, pH 7.4) to obtain LNP/DHA/CQ (LNP/DC). Furthermore, the RGD peptide-modified LNPs were obtained using the post-insertion method. A DSPE-PEG_2000_-cRGD ligand was coated on the surface of LNP/DHA/CQ in a water bath at 60°C for 30 min to obtain RLNP/DC.

### Characterization of DHA/CQ-Loaded LNPs

NanoBrook 90Plus PALS (Brookhaven, New York, United States) was used to determine the diameter, polydispersity index (PDI), and zeta potential of the prepared LNPs after 10-fold dilution with PBS (0.01 M, pH 7.4). The ROS and pH sensitivity of the LNPs were investigated by measuring the size variation under different media with varying ROS levels and pH values. LNP/DC was 10-fold diluted with PBS (0.01 M, pH 7.4/6.8/5.5) or PBS (0.01 M, pH 7.4 and containing 50/200 µM-H_2_O_2_) and incubated for 3 h. The morphology of LNP/DC was observed by transmission electron microscopy (TEM, Tecnai 12, Philips, Holland) using the negative staining method. Samples were absorbed into copper grids and stained with 1% phosphotungstic acid. The grids were observed at an acceleration voltage of 120 kV, and images were captured with a charge-coupled device (CCD) camera (Gatan 792, Gatan Inc., Pleasanton, CA, United States).

### Drug Loading Capacity and Loading Ratio

The concentrations of encapsulated DHA and CQ were determined by HPLC (LC-16, Shimadzu, Suzhou, China) with a UV detector (SPD-16, Shimadzu, Suzhou, China). LNPs were dissolved in acetonitrile and sonicated for 20 min to release the drugs. The mobile phase used were water:acetonitrile (40:60, *v*/*v*) for DHA and 7.2 mM sodium hexanesulfonate solution (containing 10 ml acetic acid and adjusted to pH 3.5 using triethylamine):acetonitrile (70:30, *v*/*v*) for CQ. Chromatographic separation was performed on a Hedera ODS-C18 column (250 × 4.6 mm, 5 ⎧m, HanBon, Jiangsu, China) at a flow rate of 1 ml/min; DHA and CQ were detected at 210 and 343 nm, respectively. The LC and LR of DHA and CQ were calculated according to Eqs. 1, 2 below.

$$LC\left(\%\right)=\frac{W_{drug\;loaded}}{W_{total\;liposome}\;}\times 100$$ (1)

$$LR\left(\%\right)=\frac{W_{drug\;loaded}}{W_{drug\;added}}\times 100$$ (2)

### *In vitro* Drug Release Behavior

The release behaviors of DHA and CQ from LNP/DC were assessed using membrane dialysis method. Different media containing pH 7.4/6.5/5.5 PBS (0.01 M) and pH 7.4 PBS (0.01 M, containing 50/200 µM-H_2_O_2_) were prepared. An aliquot of 0.5% Tween 80 was added to each medium to solubilize DHA. Dialysis bag (MWCO, 14 kDa) holding 2 ml of LNP/DC was placed into a shaking water bath containing 15 ml of medium at 37°C. At pre-set time intervals, 500 μL of medium was withdrawn and replenished with the same volume of fresh medium. The concentrations of DHA and CQ were determined using HPLC.

### Cytotoxicity and Colony Formation Assay

The cytotoxicity of DHA, CQ, DHA+CQ, LNP/DC, RLNP/DC, and RLNP in HCT116, SW480 and NCM460 cells was evaluated using the Cell Counting Kit-8 (CCK-8, Beyotime, Shanghai, China). HCT116 cells were seeded at a density of 1 × 10^4^ cells/well in 96-well plates overnight. The medium was replaced with the test preparations and cells were co-incubated for 24 h. For cell viability assay, 100 μL of serum-free medium (containing 10 μL of CCK-8) was added to each well and incubated for 1 h. The optical density (OD) of each well was measured at 450 nm using a microplate reader (Infinite M200 Pro, Tecan, Männedorf, Switzerland). The inhibition rate (%) of the test preparations was calculated using Eq. 3 below.

$$Inhibition\;rate\;\left(\%\right)=\left(1-\frac{OD_{sample}-OD_{control}}{OD_{normal}-OD_{control}}\right)\times 100$$ (3)

Colony formation assay was used to further evaluate the inhibition effect on cell proliferation. HCT116 cells were seeded in 6-well plates at 2 × 10^3^ cells/well for 24 h. DHA, CQ, DHA+CQ, LNP/DC, RLNP/DC were co-incubated with the cells at an equivalent concentration of 10 μM-DHA, and 7.5 μM-CQ, as well as blank carrier RLNP. After 24 h co-incubation, the medium was replaced and cultured for another 6 days. The colony of cells was fixed by methanol and stained with crystal violet solution. The colonies were observed and counted under microscopy.

### *In vitro* Cellular Uptake of Fluorescein-Encapsulated LNPs

The cellular uptake of LNPs was determined using calcein and cyanine-5.5 (Cy5.5)-encapsulated LNP (LNP/Cal/Cy) and RLNP (RLNP/Cal/Cy) and observed using confocal microscopy (CLSM, LSM800, Carl Zeiss Meditec AG, Jena, Germany). HCT116 cells were seeded at a density of 5 × 10^4^ cells/well and incubated overnight. The tumor cells were incubated with 2 ml serum-free medium containing free calcein and Cy5.5, LNP/Cal/Cy, and RLNP/Cal/Cy (at an equivalent concentration of 1 μg/ml calcein and 0.9 μg/ml Cy5.5). After 4 h of incubation, the cells were rinsed five times with ice-cold PBS. Then, the cells were fixed with 1 ml immunostaining fixative for 30 min and incubated with Hoechst 33342 (Sigma-Aldrich, St. Louis, MO, United States) for another 30 min. The samples were observed using confocal microscopy, and images were collected at excitation/emission maxima (Ex/Em) of 493/514 nm and Ex/Em of 675/695 nm.

### ROS Level Detection

Intracellular ROS levels were detected using the Cellular ROS Assay Kit (Cell Signaling Technology, Boston, MA, United States) following the manufacturer’s protocol. Briefly, HCT116 cells were cultured with DHA, CQ, DHA+CQ, LNP/DC, RLNP/DC (at an equivalent concentration of 10 μM-DHA and 7.5 μM-CQ), and RLNP for 12 and 24 h. The cells were then washed and incubated with 2′–7′dichlorofluorescin diacetate (DCFH-DA, 10 μM) in serum-free culture medium for 30 min at 37°C in the dark. ROS generation was quantified by fluorescence-activated flow cytometry technique (DxFLEX, Beckman Coulter, United States) that measures fluorescence intensity.

### Apoptosis Detection

The apoptosis of HCT116 cells was detected using an Annexin V-FITC/propidium iodide (PI) Apoptosis Detection Kit (Beyotime Biotech Co., Ltd., China). HCT116 cells were seeded in a 6-well plate at a density of 1 ×10^5^ cells/well for 12 h before co-incubation with DHA, CQ, DHA+CQ, LNP/DC, RLNP/DC (at an equivalent concentration of 10 μM-DHA and 7.5 μM-CQ), and RLNP. After 24 h of co-incubation, HCT116 cells were harvested and resuspended in 200 μL of buffer (containing 5 μL of Annexin V-FITC and 5 μL of PI) and incubated in the dark for 15 min. Further, the binding buffer (approximately 500 μL) was added for determination by flow cytometry (DxFLEX).

### Analysis of Cell Cycle Arrest

To determine the effect of DHA and CQ on cell cycle of colorectal cancer cells, HCT116 cells were seeded in a 6-well plate at a density of 1 ×10^5^ cells/well for 12 h before co-incubation with DHA, CQ, DHA+CQ, LNP/DC, RLNP/DC (at an equivalent concentration of 10 μM-DHA and 7.5 μM-CQ), and RLNP. After 24 h, the cells were treated with RNase A (50 µg/ml) and stained with PI (KeyGEN BioTECH, China) according to the manufacturer’s protocol. The suspension was analyzed by flow cytometry (DxFLEX).

### Transwell Invasion Assay and Wound Healing Assay

The inhibitory effects of DHA and CQ on invasion and migration of HCT116 cells were evaluated by the transwell assay and wound healing assay. For transwell invasion assay, HCT116 cells at a density of 1 ×10^6^ were cultured in the Transwell upper chambers (24-well insert; pore size 8 μm, Corning, United States) with 50 ml Matrigel-coated membranes (BD Biosciences, San Jose, CA, United States). After incubating with DHA, CQ, DHA+CQ, LNP/DC, RLNP/DC (at an equivalent concentration of 0.4 μM-DHA and 0.3 μM-CQ), and RLNP for 12 h, a cotton swab was used to remove the remaining cells; the invasive cells attached to the lower surface of the membrane were fixed with 4% paraformaldehyde (PFA) for 20 min. After staining with crystal violet for 20 min, the cells were counted under a microscope in six randomly selected fields.

For the wound healing assay, HCT116 cells were seeded in 6-well plates at a density of 1×10^6^ cells/well and cultured until 90–95% confluent. Scratch wounds were made as straight lines through the cell layer with a 200 μL pipette tip, and the cells were incubated with DHA, CQ, DHA+CQ, LNP/DC, RLNP/DC (at an equivalent concentration of 10 μM-DHA and 7.5 μM CQ), and RLNP. Images were captured at 0 and 24 h using an inverted microscope (Eclipse Ti-U, Nikon, Japan). The wound healing rate was calculated according to Eq. 4 below.

$$Wound\;healing\;rate\;\left(\%\right)=\left[1\;-\frac{area\;without\;tumor\;cells\;at\;24\;h}{area\;without\;tumor\;cells\;at\;0\;h}\right]\times 100$$ (4)

### Biochemical analysis

Biochemical analysis was performed in both cell lysates and tumor tissue homogenates. After 24 h of incubation with DHA, CQ, DHA+CQ, LNP/DC, and RLNP/DC (at an equivalent concentration of 10 μM-DHA and 7.5 μM-CQ), the cells were harvested and lysed with cell lysis buffer. Tumor tissue homogenates were isolated from mice at the experimental endpoint of the *in vivo* efficacy study and diced into small pieces for cell lysis. Malondialdehyde (MDA) and glutathione (GSH) concentrations, and superoxide dismutase (SOD) activity were measured using the MDA, GSH, and SOD kits (Nanjing Jiancheng Bioengineering Institute, Nanjing, China), respectively.

### Western Blotting

The cell lysates of “***Biochemical analysis***” were clarified by centrifugation at 12,000 × *g* at 4°C for 20 min. BCA protein assay kit (Beyotime, Institute of Biotechnology, Jiangsu, China) was used to determine the total protein concentration. Similar amounts (20–30 μg) of protein in each group were separated by 10% sodium dodecyl sulphate–polyacrylamide gel electrophoresis (SDS-PAGE) and transferred onto PVDF membranes (Millipore, Bedford, MA, United States), which were blocked using 5% bovine serum albumin (BSA) at room temperature for 1.5 h. The membrane was incubated overnight with anti-Beclin 1 antibody (ab210498, Abcam, Cambridge, United Kingdom), anti-LC3B antibody (MAB85582, R&D Systems, Minneapolis, United States), anti-p62 antibody (ab109012, Abcam), anti-β-actin antibody (ab8227, Abcam), anti-VEGFA antibody (ab52917, Abcam), anti-Angiopoietin 1 antibody (ab183701, Abcam), and anti-Paxillin antibody (ab23510, Abcam) at 4°C. After washing, the membranes were incubated for 2 h at room temperature with horseradish peroxidase-conjugated Goat anti-Rabbit IgG antibody (H&L) and horseradish peroxidase-conjugated Rabbit anti-Mouse IgG antibody (H&L) (Abcam), and an enhanced chemiluminescence (ECL) reagent (Thermo Fisher Scientific, Pittsburgh, PA, United States). Digital images of the blots were obtained using the Syngene Gel Imaging System (Tanon 6600, Tanon, Shanghai, China) and analyzed using the Image Lab Software. Total proteins from the tumor tissue homogenates were quantified using the BCA protein assay kit. Equal amounts (30 μg/10 µL) of protein in each group were separated by 10% SDS-PAGE and transferred to PVDF membranes (Millipore). The processing procedure used was the same for cell samples, as described above.

### *In vivo* Anti-Metastasis and Anti-Tumor Activity

Six-week-old male BALB/c nude mice (18–22 g) were supplied by the Comparative Medicine Centre of Yangzhou University. Mice were maintained under standard specific-pathogen-free (SPF) conditions (temperature of 23 ± 2°C and relative humidity of 45 ± 10%). Animal studies were approved by the Animal Welfare and Ethics Committee of Guizhou Medical University (approval no. 1900117). A mouse model of orthotopic metastasis of colorectal cancer was established according to the previous report (Chen et al., 2020), and successful establishment of the model was confirmed by the liver metastatic nodes on the appearance and the histological analysis in the pre-experiment. HCT116 cells (2 × 10^6^) were injected into the subserous layer of the cecum in mice after laparotomy. After 30 days of injection, nude mice were randomly divided into PBS, DHA+CQ, LNP/DC, RLNP/DC, and blank RLNP groups (*n* = 6). Free drugs and LNP solution (at an equivalent dose of 4 mg/kg DHA and 5.5 mg/kg CQ) were intravenously injected via the tail vein every alternate day for five times in total. The same volume of PBS was injected in the control group. The mice were sacrificed on day 60, and their intestine and liver were separated. The tumor volume (V) was assessed by measuring the largest diameter (L) and the smallest diameter (W) with a Vernier caliper using the following equation:

$$V=\frac{1}{2\;}\times L\times W^{2}$$ (5)

For evaluation of anti-metastasis activity, histological analysis of the liver was performed after hematoxylin-eosin (H&E) staining, and the liver metastatic nodes were quantified. The body weight of mice was recorded every 6 days until the end of the experiment, and the survival rate was also estimated.

### Statistical Analysis

Data are presented as the mean ± standard deviation (SD) of at least three independent experiments. Significant differences between two groups were identified using the student’s t-test; one-way ANOVA followed by Tukey’s post hoc test was used for more than three groups. Statistical significance was set at *p* < 0.05. *In vivo* survival analysis was performed using the Kaplan-Meier method, and a log-rank test was performed to analyze the differences among the survival curves.

## Results and Discussion

### Preparation and Characterization of DHA and CQ Co-Encapsulated LNPs

**FIGURE 1:**
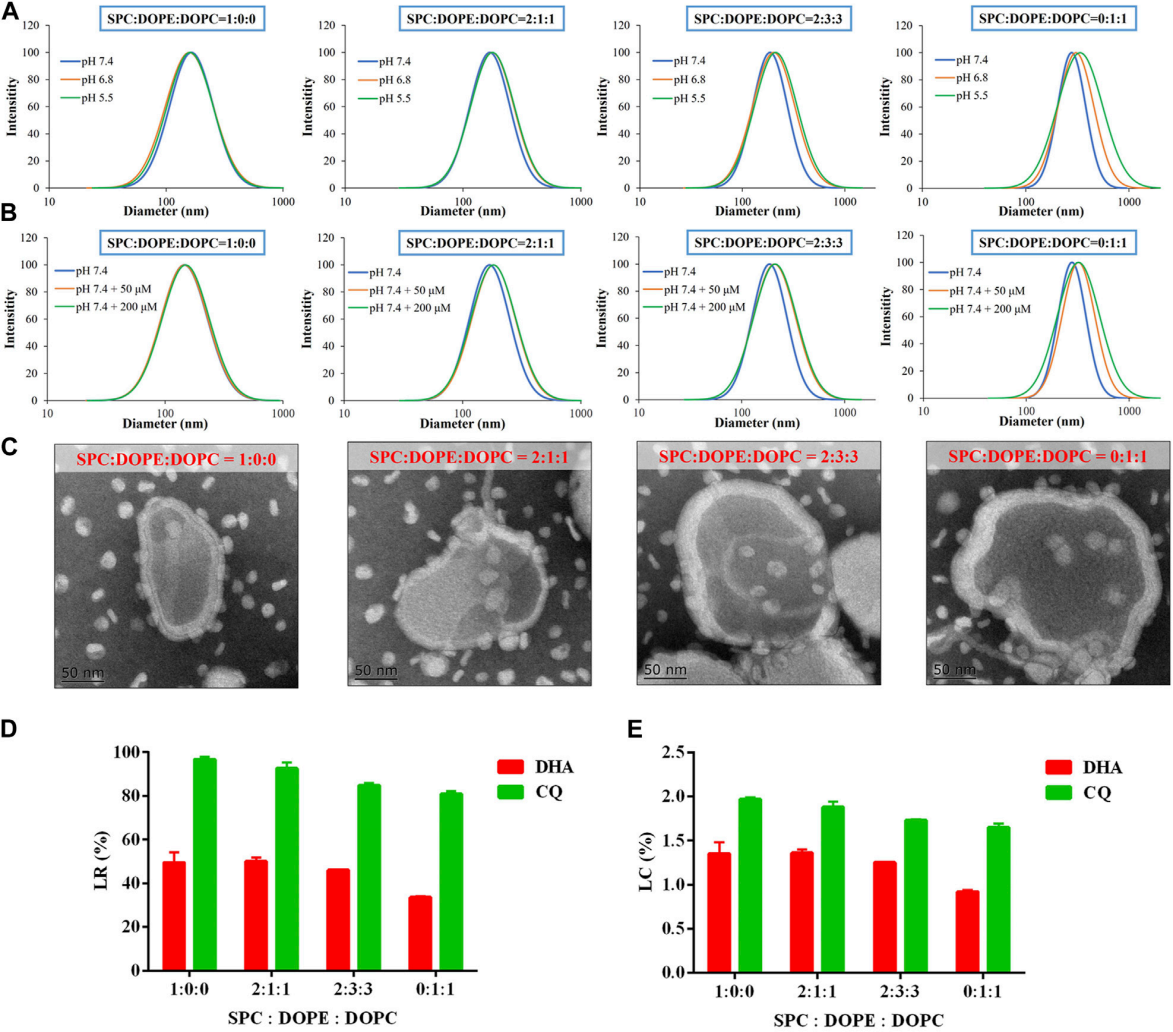
Preparation and optimization of LNP/DC with respect to SPC/DOPE/DOPC ratio. **(A, B)** Size distribution of LNP/DC prepared at various weight ratio of SPC/DOPE/DOPC in pH 7.4, 6.8, 5.5 **(A)** and pH 7.4 + 50/200 μM-H_2_O_2_ **(B)** medium. **(C)** TEM images of LNP/DC prepared at various weight ratio of SPC/DOPE/DOPC (Scar bar reads 50 µm). **(D)** LR and **(E)** LC of DHA and CQ for LNP/DC prepared at various weight ratio of SPC/DOPE/DOPC. Each bar represents the mean ± SD (*n* = 3).

LNPs composed of mixed phospholipids (SPC, DOPE, and DOPC), CO, and GT were developed in this study for co-delivery of DHA and CQ. LNPs were optimized for SPC/DOPE/DOPC ratio by determining its size under pH 7.4 (physical environment), pH 6.8 (tumor microenvironment), pH 5.5 (lysosome environment), and pH 7.4 + 50/200 µM-H_2_O_2_ (intracellular high level of ROS), and for encapsulation efficiency of DHA and CQ. The sensitivity of LNP/DC to pH (Figure 1A) and ROS (Figure 1B) was evaluated based on size distribution of the nanoparticles; the size distribution curve of LNP/DC moved towards right as the proportion of DOPE and DOPC increased, indicating the formation of pH/ROS dual sensitive LNP/DC with the addition of DOPE and DOPC. Inclusion of DOPE preferred transformation from bilayer to hexagonal phase, particularly under acidic conditions, owing to the small head group and large tail and formation of intermolecular hydrogen bonding between head groups, demonstrating pH sensitivity (Litzinger and Huang, 1992). Inclusion of DOPC and DOPE resulted in formation of a heterogeneous lipid bilayer that interfered with the stability of the membrane bilayer formed by SPC alone, which might be attributed to the unsaturated bonds incorporated in these synthetic phospholipids and phase transformation potential of DOPE.

Moreover, morphology of LNP/DC prepared at various SPC/DOPE/DOPC ratios was observed by TEM. As shown in Figure 1C, the size of LNP/DC ranged from approximately 170–240 nm at pH 7.4, which was in agreement with the measurement by dynamic light scattering (Figures 1A,B). The lipid bilayer of LNP/DC ranged from 15 to 25 nm and inclusion of GT was clearly observed. Moreover, thickness of the layer increased as the proportion of DOPE and DOPC increased. However, reduced drug loading for DHA and CQ was observed as the proportion of DOPE and DOPC increased (Figures 1D,E), suggesting that thicker lipid layer did not favor of DHA encapsulation. The LR of DHA decreased to less than 35% when LNP/DC was prepared using only DOPE and DOPC, indicating that SPC favors DHA encapsulation. Contrarily, the LR of CQ was higher than 80% regardless of the ratio of SPC/DOPE/DOPC, which was attributed to the inherent high efficiency of the remote loading method of drug encapsulation. Although swollen particles were observed as the proportion of DOPE and DOPC increased, the extensive internal water phase did not contribute to the encapsulation of CQ as hypothesized that thicker lipid bilayer might prevent remote loading of CQ. LNPs were prepared for further experiments using SPC/DOPE/DOPC at a ratio of 2:3:3, which was optimized based on particle size, pH/ROS dual sensitivity and drug loading capability.

**FIGURE 2:**
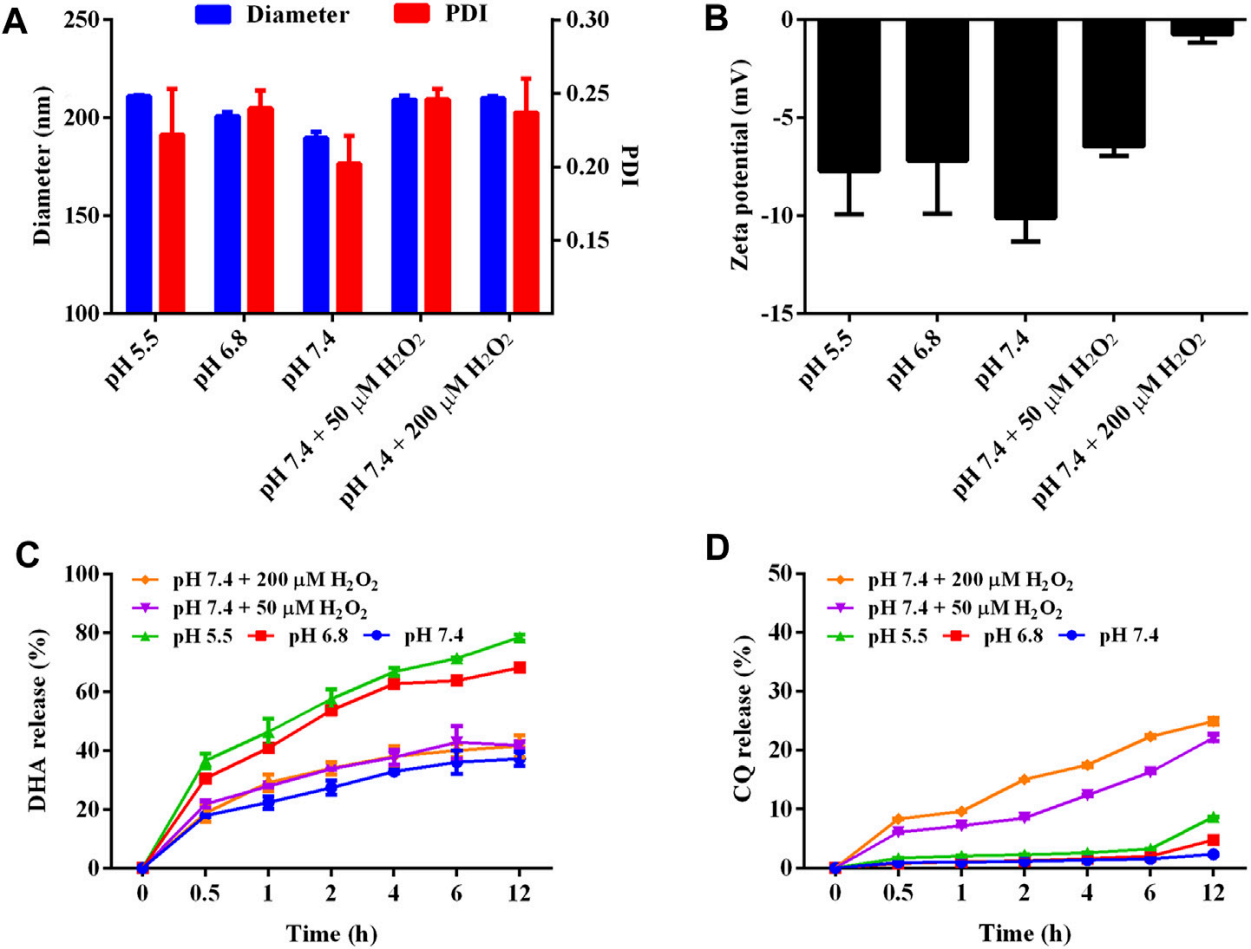
Characterization on the pH and ROS dual-sensitivity of LNP/DC. **(A, B)** Diameter, PDI **(A)** and zeta potential **(B)** of LNP/DC in different medium. **(C, D)** Drug release behavior of DHA **(C)** and CQ **(D)** from LNP/DC in different mediums.

Moreover, pH/ROS sensitivity of LNP/DC was confirmed by variation in particle size, PDI, and zeta potential (Figures 2A,B). Particles were ranged from 190 to 210 nm without any change in PDI with decrease in pH and H_2_O_2_ addition. The zeta potential of LNP/DC was similar at different pH values, however, it changed from −10 to −5 mV and neutral in the presence of 50 μM-H_2_O_2_ and 200 μM-H_2_O_2_, respectively. This might be because of breakage of fatty chains of phospholipids, which interfered with the stability and integrity of the lipid layer. Moreover, the release behavior of DHA and CQ from LNP/DC showed their differing sensitivity to pH and ROS (Figures 2C,D). DHA was more sensitive to acidic environment and exhibited faster release at pH 5.5, whereas CQ was more sensitive to ROS level. These findings suggest that the phase transformation of DOPE in an acidic environment diminished the interaction between DHA and the lipid layer, which facilitated the release of DHA, whereas integrity of the lipid bilayer was maintained, which hindered the release of CQ from the interior phase. Although high level of ROS was reported to induce oxidation of unsaturated fatty acids in lipids and thereby to enhance the hydrophilicity of the lipid bilayer, however, it might not be strong enough to interfere with the interaction between DHA and lipids due to lack of ROS sensitivity of DHA. In contrast, the enhanced hydrophilicity of the lipid bilayer promoted the release of CQ despite less than 30% cumulative release detected at 12 h. Thus, LNP/DC with pH/ROS dual sensitivity was successfully prepared.

Furthermore, RLNP/DC was prepared from DSPE-PEG-RGD and LNP/DC using the post-insertion method. Blank LNP, LNP/DC, and RLNP/DC were characterized as shown in Table 1. Drug encapsulation increased the size of LNP/DC, which might have resulted from incorporation of DHA into the lipid bilayer. DEPE-PEG-RGD insertion increased the size only slightly but reduced the zeta potential considerably from −10 to −5 mV, indicating that RLNP/DC was successfully prepared. The stability of LNP/DC and RLNP/DC was investigated by the size variation under storage at 4°C (Supplementary Figure S2). LNPs exhibited slight size increasement from around 200 to 240 nm and small range fluctuation of PDI between 0.2 and 0.3, indicating preferable stability of LNP/DC and RLNP/DC for at least 1 week at 4°C.

**TABLE 1:** Properties of DHA and CQ-loaded LNPs.

Samples	Characteristics		
	Diameter (nm)	PDI	Zeta potential (mV)
LNP	170.50 ± 3.30	0.199 ± 0.014	−10.07 ± 0.11
LNP/DC	189.78 ± 3.06	0.200 ± 0.020	−10.11 ± 1.20
RLNP/DC	195.36 ± 1.69	0.240 ± 0.010	−5.05 ± 0.76

Each data represents the mean ± SD of at least three independent experiments.

### Cellular Uptake and Cytotoxicity Assay

**FIGURE 3:**
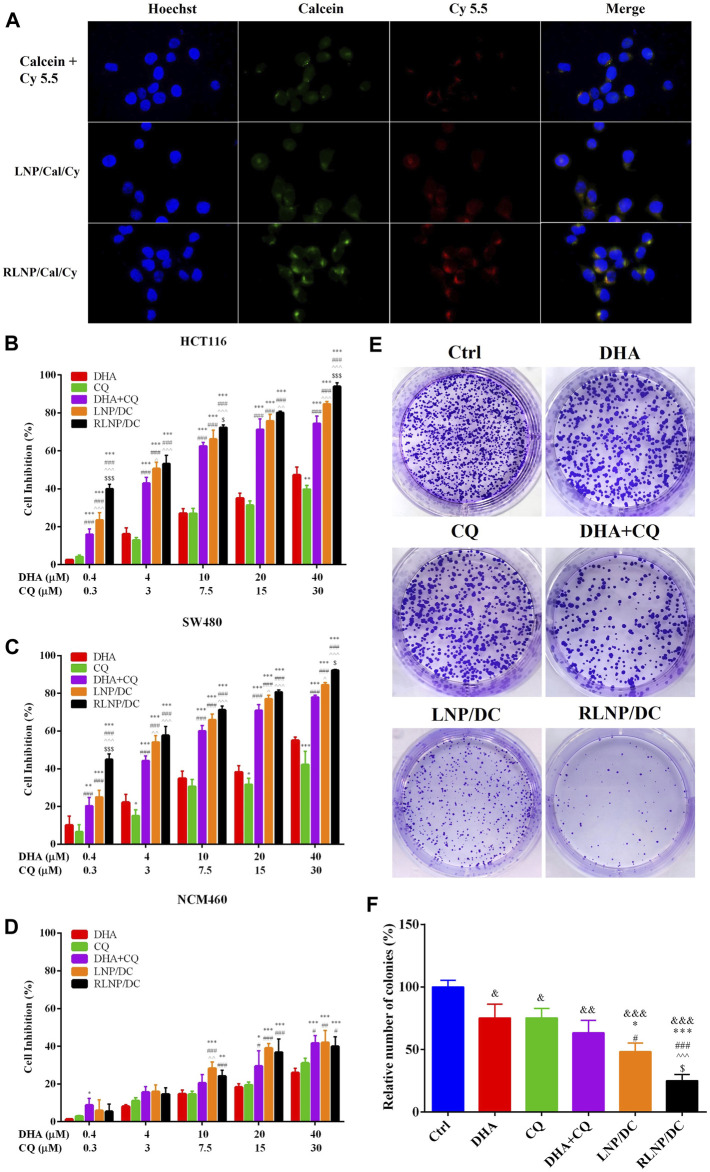
Evaluation of cytotoxic effect and cellular uptake of LNPs. **(A)** Cellular uptake of calcein and Cy5.5-labeled LNPs by HCT116 colorectal tumor cells after 4 h of treatment (Scar bar reads 20 µm). **(B , D)** Cytotoxicity of DHA, CQ, DHA + CQ, LNP/DC, and RLNP/DC on HCT116 cells **(B)**, SW480 cells **(C)** and NCM460 cells **(D)**. **(E, F)** Colony formation assay via observation **(E)** and quantification **(F)** by microscopy. Each bar represents the mean ± SD (*n* ≥ 3). ^*^ *p* < 0.05, ^&^ *p* < 0.05, ^&&^ *p* < 0.01, and ^&&&^ *p* < 0.001 vs. Ctrl; ^**^ *p* < 0.01 and ^***^ *p* < 0.001 vs. DHA; ^#^ *p* < 0.05 and ^###^ *p* < 0.001 vs. CQ; ^^^ *p* < 0.05, ^^  ^ *p* < 0.01, and ^^  ^  ^ *p* < 0.001 vs. DHA + CQ; ^$^ *p* < 0.05 and ^$$$^ *p* < 0.001 vs. LNP/DC.

We prepared and characterized the hydrophobic fluorescein-Cy5.5 and hydrophilic fluorescein-calcein co-encapsulated LNPs for investigation of the cellular uptake by HCT116 cells (Supplementary Figure S2). In the images taken by the confocal microscopy, fluorescence of Cy5.5 and calcein were marked by the red and green colors, respectively (Figure 3A). LNPs group exhibited higher fluorescence intensity than the free fluorescein combination groups. The overlap of Cy5.5 and calcein in RLNP/Cal/Cy group indicated by the yellow color was the brightest among all groups. This result suggested that LNPs, particularly RLNPs, could promote the cellular uptake of encapsulated hydrophilic and hydrophobic fluorescein compounds. Conclusively, RLNP/DC has great potential to facilitate the accumulation of encapsulated DHA and CQ in HCT116 cells to realize their synergistic effects on inhibition of cell growth.

To evaluate the potential therapeutic effect of DHA and CQ combination therapy in colorectal cancer, we measured *in vitro* cytotoxic effects of free drugs and drug-loaded LNPs in HCT116 and SW480 cells. The synergistic antitumor effect of the drug combination was demonstrated, and the optimal ratio of DHA and CQ was detected on HCT116 cells (Supplementary Figure S1). The combination index (CI) values ranged from 0.20 to 0.49 at different molar ratios, confirming the strong synergistic effect of DHA and CQ combination therapy at a ratio of 4:3 (mol/mol). Therefore, during preparation of LNP/DC, we added DHA and CQ in definite amounts to obtain a final preparation with DHA/CQ at a molar ratio of 4:3 after adjusting for LR. Cytotoxic effects of free drug, free drugs combination, LNP/DC, and RLNP/DC in HCT116 cells (Figure 3B), SW480 cells (Figure 3C), and NCM460 cells (Figure 3D) were measured. Blank RLNP exhibited no cytotoxicity in all cell lines (Supplementary Figure S4). DHA and CQ combination therapy exhibited considerably enhanced cell inhibitory effect in both HCT116 and SW480 cells compared with free single drugs. LNP/DC showed slightly improved cytotoxicity as compared to the free drugs combination, however significant cytotoxic effect was observed in RLNP/DC. Furthermore, the enhanced anti-proliferation effect of DHA and CQ combination by LNPs to HCT116 cells was verified by the significantly compromised colony formation capacity, especially for RLNP/DC (Figures 3E,F). Considering the optimal cellular uptake of RLNP by HCT116 cells, the enhanced cytotoxicity of RLNP/DC might be directly related to the effective co-delivery of DHA and CQ. In contrast, both DHA and CQ exhibited markedly lower cytotoxic effect in NCM460 cells, which was slightly enhanced at high concentrations of DHA and CQ in combination. However, LNPs did not show further increase in the cytotoxicity of DHA and CQ combination. Thus, selective cytotoxic effect of DHA, CQ, and drug loaded-LNPs in human colon carcinoma cells was observed.

According to the cell inhibitory mechanism, Fe^2+^ plays an important role in the activation of DHA to generate ROS (Shen et al., 2020). Considering proliferating tumor cells are highly in need of Fe^2+^ compared with normal cells (Shterman et al., 1991), it may partly contribute to the preferential cytotoxicity of DHA towards colorectal cancer cells to normal colonic epithelial cells. In addition, autophagy plays a vital role in the progression of tumor cells compared to normal cells to remove the oxidized metabolic products that might induce DNA damage (Cicchini et al., 2015). RGD-modified nanocarrier did not exhibit improved intracellular drug delivery in NCM460 cells that were not overexpressing integrin receptors, defining the lack of difference in cytotoxicity between the free drug combination and LNPs. Collectively, DHA and CQ combination therapy has great potential to suppress the growth of colorectal cancer cells along with low toxicity, and RLNP showed further increase in the anti-proliferative activity.

### *In vitro* Evaluation on ROS Regulation and Autophagy Inhibition

**FIGURE 4:**
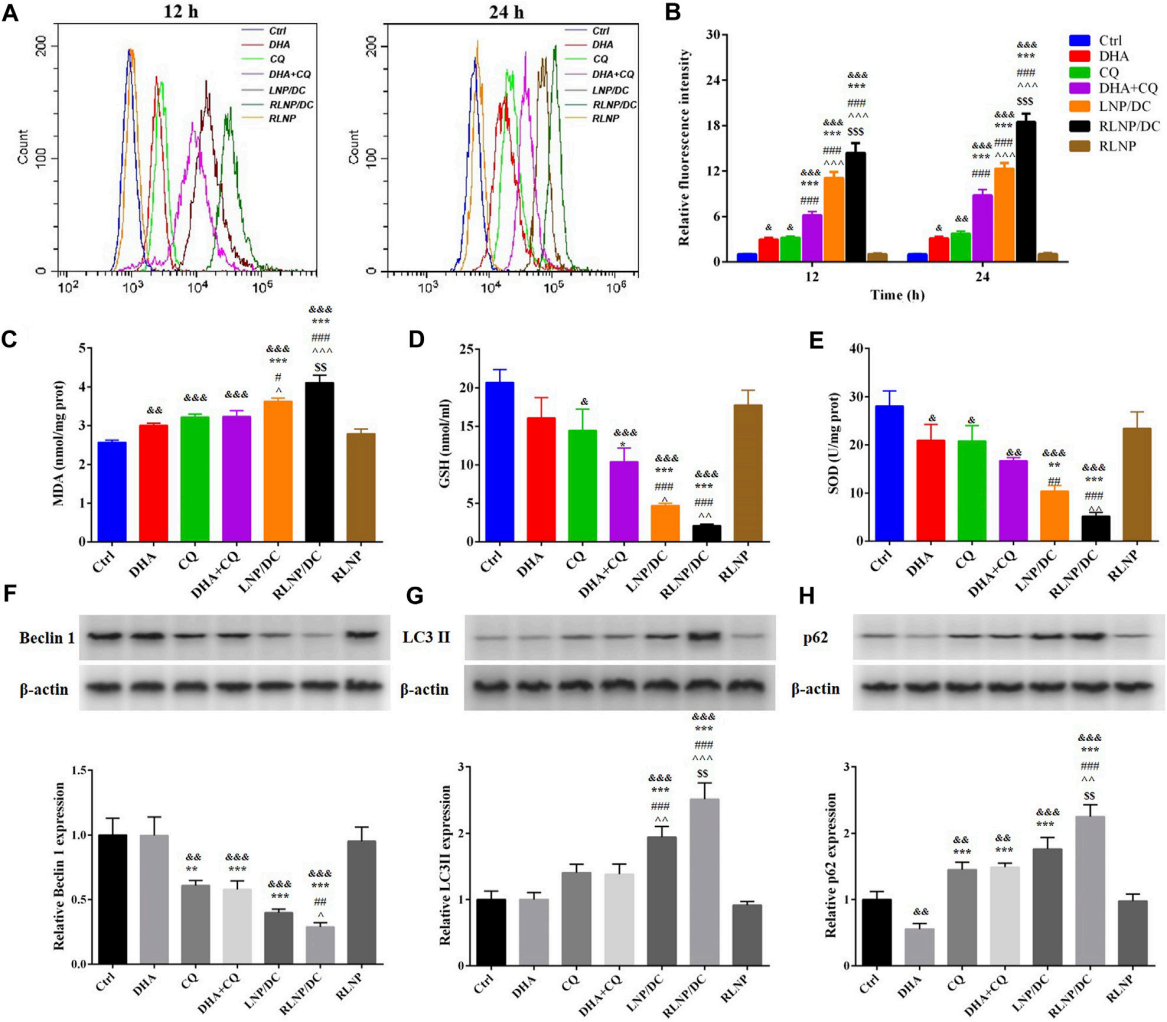
Regulation of the intracellular ROS level and the underlying mechanism. **(A, B)** Quantitation of intracellular ROS level of HCT116 cells after 12 and 24 h of treatment by DHA, CQ, DHA + CQ, LNP/DC, RLNP/DC, and RLNP. **(C–E)** The level of MDA and GSH, and SOD activity after 24 h of treatment. **(F, H)** Expression level of autophagy-related proteins including Becilin 1, LC3 II, and p62 after 24 h of treatment. Each bar represents the mean ± SD (*n* = 3). ^&^ *p* < 0.05, ^&&^ *p* < 0.01, and ^&&&^ *p* < 0.001 vs. Ctrl; ^*^ *p* < 0.05, ^**^ *p* < 0.01, and ^***^ *p* < 0.001 vs. DHA; ^#^ *p* < 0.05, ^##^ *p* < 0.01, and ^###^ *p* < 0.001 vs. CQ; ^^^ *p* < 0.05, ^^  ^ *p* < 0.01, and ^^  ^  ^ *p* < 0.001 vs. DHA + CQ; ^$$^ *p* < 0.01 and ^$$$^ *p* < 0.001 vs. LNP/DC.

The major mechanism underlying the synergistic antitumor effect of DHA and CQ combination therapy is the regulation of ROS levels. As previously reported, ROS generated by the cleavage of the endoperoxide bridge of ART and its analogs plays an important role in inhibiting the growth of tumor cells (Mercer et al., 2007). CQ, an autophagy inhibitor, could further elevate the intracellular ROS level via preventing the formation of autolysosome (Redmann et al., 2017). In HCT116 cells, intracellular ROS levels were detected after treatment for 12 and 24 h (Figures 4A,B), which revealed similar elevation effects of DHA and CQ. Higher level of ROS was detected in the free drug combination group than in single drug groups, which was further enhanced by LNPs. Consistent with the cytotoxicity assay results, RLNP/DC-treated cells showed the highest ROS levels among all groups. No obvious time-dependent effect on ROS accumulation was observed in any of the groups, suggesting that ROS elimination mechanism is accompanied by ROS generation (Espinosa-Diez et al., 2015). Meanwhile, oxidative stress markers, including small molecules and antioxidant enzymes, were detected after treatment (Figures 4C–E). The levels of MDA and GSH, and SOD activity further revealed the antioxidant response induced by ROS generation. Accumulation of MDA and downregulation of GSH synthesis and SOD activity were observed in all groups except RLNP. In agreement with ROS levels in different groups, the free drug combination and LNP groups exhibited high MDA accumulation and low levels of antioxidant enzymes.

Moreover, autophagy-related proteins were analyzed by western blotting (Figures 4F–H). CQ-induced autophagy inhibition was confirmed by downregulation of Beclin 1 protein expression and upregulation of LC3II and p62 protein expressions, indicating hinderance in the initiation of autophagy and formation and degradation of autophagosomes, respectively (Saha et al., 2018). The free drug combination group also showed the similar findings. Moreover, RLNP/DC showed the most significant regulatory effects on autophagy-related protein expressions. It is reasonable to propose that RLNP/DC enhanced ROS generation via effective intracellular co-delivery of DHA and CQ, thereby leading to optimal inhibitory effects on the growth of colorectal cancer cells.

In response to organelles damage caused by oxidative stress, autophagy is activated to recycle intracellular materials, which leads to either improved cell growth or cell death (Cicchini et al., 2015). Autophagy plays an important role in recycling of Fe^2+^, which is critical for the iron-dependent generation of ROS by DHA (Lee et al., 2012). Therefore, a possible paradoxical effect of autophagy inhibition is that it contributes to the irreversible upregulation of ROS levels, but partly counteracts the cytotoxicity of DHA by reducing iron viability. In our study, the net result was a significant increase in ROS level by DHA and CQ combination therapy. This could be attributed to the high concentration of Fe^2+^ in HCT116 cells, which is sufficient for DHA activation and high dependence of tumor cells on autophagy for proliferation. Although DHA-induced autophagy contributed to non-apoptotic cell death in cancer treatment (Hu et al., 2014; Chen et al., 2015), it was not observed in HCT116 cells treated with DHA alone.

### Cell Cycle Arrest Analysis and Apoptosis Rate Detection

**FIGURE 5:**
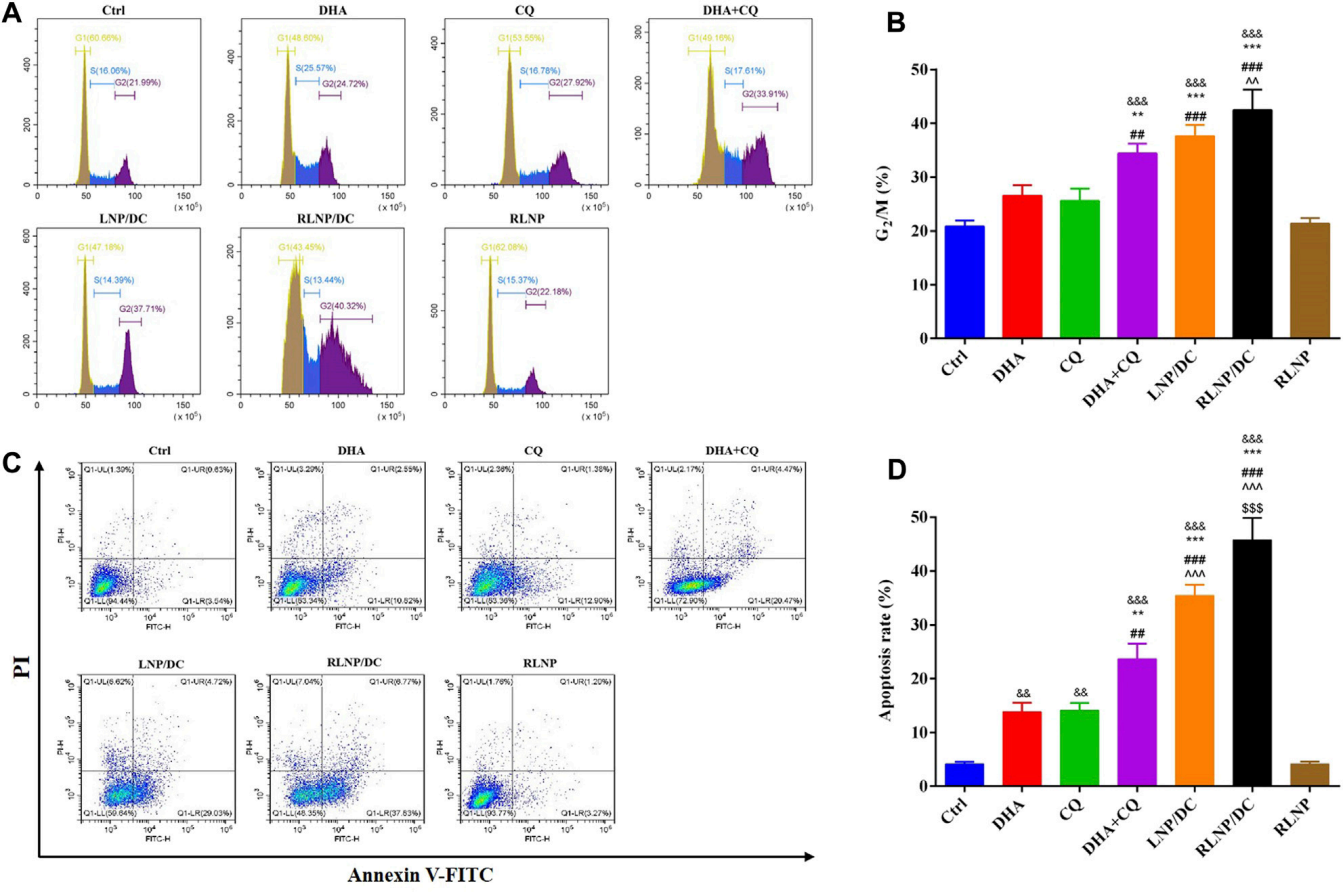
Analysis of cell-cycle arrest **(A, B)** and apoptosis rate **(C, D)** assay in HCT116 cells after 24 h of treatment by DHA, CQ, DHA + CQ, LNP/DC, RLNP/DC, and RLNP. Each bar represents the mean ± SD (*n* = 3). ^&&^ *p* < 0.01 and ^&&&^ *p* < 0.001 vs. Ctrl; ^**^ *p* < 0.01 and ^***^ *p* < 0.001 vs. DHA; ^##^ *p* < 0.01 and ^###^ *p* < 0.001 vs. CQ; ^^  ^ *p* < 0.01 and ^^  ^  ^ *p* < 0.001 vs. DHA + CQ; ^$$$^ *p* < 0.001 vs. LNP/DC.

ART and its analogs have been reported to arrest the cell cycle by interfering with the expression and activity of regulatory enzymes in cell proliferation (Zhang et al., 2012; Chen et al., 2014; Lin et al., 2016). As shown in Figures 5A,B, G_2_/M cell cycle arrest in HCT116 cells after treatment with single drug was clearly increased, although no significant difference was detected. In contrast to previous reports that DHA exhibited G_0_/G_1_ cell cycle arrest in HCT116 cells, DHA induced S phase arrest in our study (Figure 5A). Cell cycle arrest induced by CQ alone and the combination was mainly attributed to ROS generation and accumulation, considering that ROS-dependent cell cycle arrest usually occurs in the G_2_/M phase. LNPs, particularly RLNP/DC, slightly improved the percentage of cells in the G_2_/M phase compared to the free drug combination. This was consistent with the ROS level in each group (Figure 4B), which further confirmed that DHA and CQ combination therapy mainly caused ROS-dependent cell cycle arrest in HCT116 cells.

High levels of ROS induce cell cycle arrest and DNA damage, eventually leading to cell death. Apoptosis has been reported as the primary mechanism of DHA-induced cell death in cancer, and both intrinsic/extrinsic and mitochondrial pathways have been proposed to be involved in DHA-induced cell death in colorectal cancer (Gao et al., 2011; Zhang et al., 2012; Liu et al., 2013). Free DHA and CQ showed similar rates of apoptotic cell death in HCT116 cells (Figures 5C,D). Higher apoptosis rate was detected in the drug combination group than in either of the single-drug groups. Most of the apoptotic cell were at the early phase of apoptosis in all the groups. The apoptosis rate of DHA+CQ group at the early phase is equal to the sum of that in the single DHA and CQ group. LNPs with the drug combination encapsulated further improved the early apoptosis rate. Intriguingly, RLNP/DC induced approximately 40% early apoptosis, which is about 2-folds of that in the free drug combination group. It might be attributed to the effective intracellular co-delivery of DHA and CQ mediated by RLNP. Taken together, DHA and CQ combination enhanced G_2_/M cell cycle arrest and apoptotic cell death via ROS level regulation, elucidating enhanced cytotoxic effect in HCT116 cells compared with single drugs. RLNP/DC further elevated the intracellular ROS level and the subsequent cascade events, attributable to the effective co-delivery of DHA and CQ.

### *In vitro* Anti-Metastasis Activity

**FIGURE 6:**
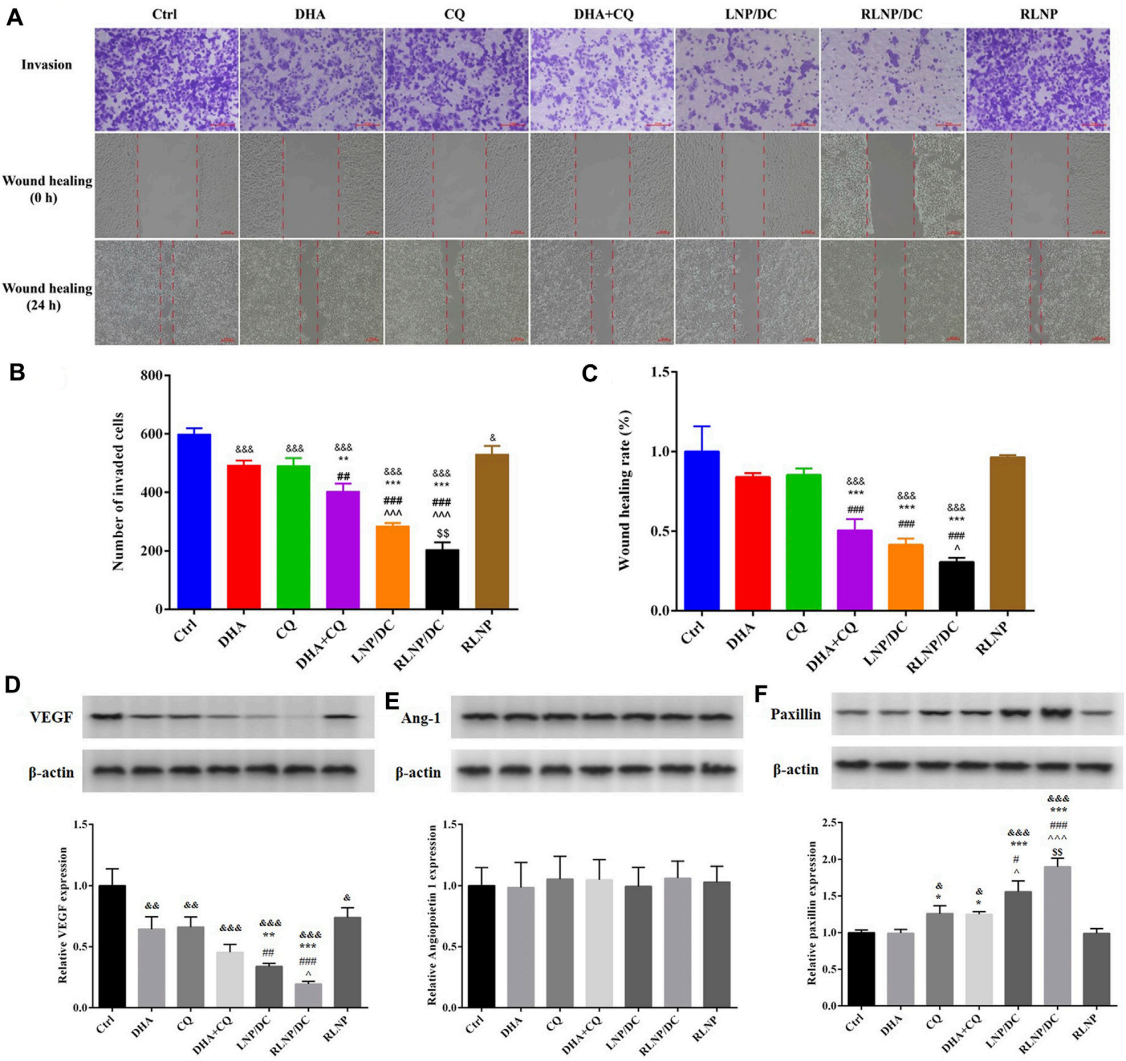
Assessment of *in vitro* anti-metastasis effect and detection of metastasis-related proteins. **(A–C)** Transwell invasion assay and wound healing rate assay in HCT116 cells after treatment by DHA, CQ, DHA + CQ, LNP/DC, RLNP/DC, and RLNP. **(D–F)** Expression level of metastasis-related proteins including VEGF, Ang-1, and Paxillin after 24 h of treatment. Each bar represents the mean ± SD (*n* = 3). ^&^ *p* < 0.05, ^&&^ *p* < 0.01, and ^&&&^ *p* < 0.001 vs. Ctrl; ^*^ *p* < 0.05, ^**^ *p* < 0.01, and ^***^ *p* < 0.001 vs. DHA; ^##^ *p* < 0.01 and ^###^ *p* < 0.001 vs. CQ; ^^^ *p* < 0.05 and ^^  ^  ^ *p* < 0.001 vs. DHA + CQ; ^$$^ *p* < 0.01 vs. LNP/DC.

DHA and CQ have been reported to possess anti-metastatic activity in various cancer cells via regulation of angiogenesis and focal adhesion-related proteins, respectively. We assessed the inhibitory effects of these drugs on invasion and metastasis of HCT116 cells using the transwell invasion assay and wound healing assays (Figures 6A–C). Single drugs inhibited the invasion and prevented wound healing of HCT116 cells slightly and to the same extent. DHA and CQ combination exhibited significantly increased inhibitory effect on cell invasion, which was further reinforced by LNPs, particularly RLNP/DC. In addition, blank carrier RLNP had no effect on the invasion and metastasis of HCT116 cells.

Previous studies have reported that DHA exerts antiangiogenic activity in various cancers, therefore, secretion of angiogenic factors VEGF and Ang-1 by HCT116 cells was determined after treatment (Figures 6D,E). Meanwhile, secretion of paxillin, which is required for focal adhesion between tumor cells and ECM, and reported as the main anti-metastasis mechanism of CQ in breast cancer, was determined (Figure 6F). Notably, no effect on Ang-1 expression was detected after treatment, though DHA and CQ downregulated the expression of VEGF protein to the same extent. Further reduction in VEGF expression was observed in the free drug combination group and LNP groups, however, the difference was significant only in the LNP groups. CQ alone upregulated the expression of paxillin, whereas no variation in the DHA-treated group and therefore no synergistic effect in the DHA and CQ combination group was observed with respect to paxillin. However, LNPs, particularly RLNP/DC, further enhanced the regulatory effect of CQ on paxillin expression. This is consistent with the results of invasion and wound healing inhibition that RLNP/DC possessed the greatest anti-metastasis effect *in vitro*.

Both angiogenesis inhibition and focal adhesion enhancement contributed to anti-metastasis activity of DHA and CQ combination therapy in colorectal cancer cells *in vitro*. Tumor neovascularization plays an important role in the aggressive growth and metastasis of tumor cells (Zetter, 1998). DHA has been reported to repress the expression of proangiogenic factors, including VEGF and VEGFR, by blocking the nuclear translocation of NF-κB and the binding between p65 and the VEGFR promoter (Wang et al., 2011; Dong et al., 2014). Moreover, autophagy was reported to promote the secretion of VEGF via several pathways. CQ being an autophagy inhibitor leads to downregulation of VEGF. In addition, the initial stage of metastasis involves detachment of tumor cells from the primary tumor lesion; this step is closely related to the focal adhesion between tumor cells and the ECM. Paxillin degradation by autophagy disrupts the “bridge” between tumor cells and the ECM, leading to detachment of cells from the ECM. However, this initial step of metastasis was impeded by CQ via autophagy inhibition. Collectively, we speculate downregulation of VEGF and upregulation of paxillin likely to be the main mechanisms underlying anti-metastasis activity of DHA and CQ combination therapy.

### *In vivo* Anti-Proliferation and Anti-Metastasis Effect on Colorectal Cancer in Mice

**FIGURE 7:**
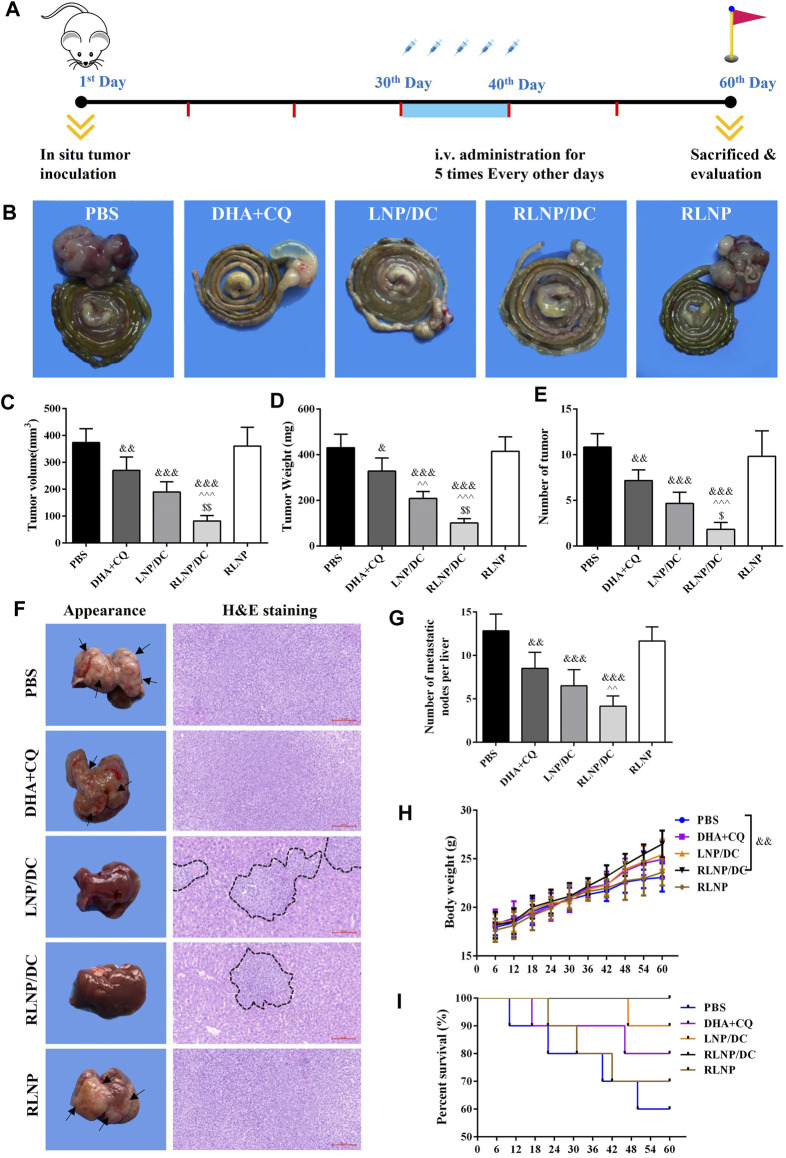
Evaluation of *in vivo* anti-proliferation and anti-metastasis effects in a mouse model of orthotopic metastasis of colorectal cancer. **(A)** Schematic illustration of *in vivo* experiments involving therapeutic regimen. **(B)** Representative images of colorectum from each group. **(C –E)** Measurement of tumor volume **(C)**, tumor weight **(D)**, and number of tumor **(E)** on day 60. **(F)** Representative images of liver and H and E staining (×200) of metastatic tissue section from each group. Arrowheads indicate the metastatic nodes on the appearance of liver and black imaginary lines mark the contractible margin of metastasis nodes. **(G)** Quantitation of the number of metastatic nodes on liver. **(H)** Body weight of the nude mice. **(I)** Survival rate of mice from each group. Each bar represents the mean ± SD (*n* ≥ 6). ^&^ *p* < 0.05, ^&&^ *p* < 0.01, and ^&&&^ *p* < 0.001 vs. Ctrl;  ^  ^ *p* < 0.01 and ^^  ^  ^ *p* < 0.001 vs. DHA + CQ; ^$^ *p* < 0.05 and ^$$^ *p* < 0.01 vs. LNP/DC.

Considering potential findings *in vitro*, anti-proliferation and anti-metastasis effects of the preparations were further investigated in a mouse model of orthotopic metastasis of colorectal cancer. A schematic illustration of *in vivo* antitumor study is shown in Figure 7A. The nude mice were inoculated with colorectal cancer cells and bred for 30 days; mice were then injected PBS, DHA+CQ, LNP/DC, RLNP/DC, and blank RLNP via tail vein every alternate day for 5 times in total and sacrificed at the day 60. All the preparations comprising DHA and CQ combination exhibited suppressive effects on tumorigenesis of colorectal cancer at the end of experiments, as indicated by the separated segments of the colorectum (Figure 7B). The anti-proliferation effect of DHA and CQ combination therapy on colorectal cancer was evaluated with respect to tumor volume, tumor weight, and number of tumors (Figures 7C–E). LNPs further enhanced the therapeutic effects of the drug combination, however, significant difference was observed in the RLNP/DC group compared with other groups.

Moreover, the effect of DHA and CQ combination therapy on prevention of liver metastasis of colorectal cancer was evaluated. Mice livers were isolated at the end of the experiments, and H&E staining was performed to identify metastatic lesion (Figure 7F). The metastatic nodes were identified by white spots on the liver surface and observed in H and E staining images. The free drug combination and drug-loaded LNPs exerted suppressive effects on liver metastasis of colorectal cancer, as indicated by the decreased number of metastatic nodes on the appearance and the contractible margin of metastatic nodes in H&E staining images. This was further confirmed by the reduced number of metastatic nodes in the liver after treatment with RLNP/DC (Figure 7G). The body weight of the mice was monitored every 6 days during *in vivo* experiment (Figure 7H). A similar upward sloping curve before the 36th day was observed in all groups, whereas a significant difference in body weight was detected between the RLNP/DC and PBS groups at the end of the experiment, indicating therapeutic effectiveness and good biocompatibility of the preparations. Eventually, all preparations comprising DHA and CQ combination improved the survival rate, difference being insignificant between all groups, and no mice died in the RLNP/DC group till the end of the experiment (Figure 7I). Collectively, anti-proliferation and anti-metastasis effects of DHA and CQ combination were confirmed in a mouse model of orthotopic metastasis of colorectal cancer. LNPs, particularly RLNP/DC, further enhanced the therapeutic effect of the drug combination in mice and showed a good safety profile.

Accumulating evidence has shown that the anti-proliferative activity of ARTs on tumor cells was enhanced by CQ via broking ARTs-induced autophagy to improve the apoptosis level in previous studies (Button et al., 2014; Ganguli et al., 2014; Chen et al., 2015). However, the synergistic effect of DHA and CQ combination against colorectal cancer was revealed only *in vitro*. Herein, we demonstrated that CQ-induced autophagy inhibition profoundly promoted DHA-induced ROS generation, leading to ROS-dependent cell cycle arrest at G_2_/M phase and elevated apoptosis rate compared with DHA alone. Moreover, anti-proliferative activity of DHA and CQ combination therapy was further evaluated in a mouse model of orthotopic metastasis of colorectal cancer. In contrast to similar cytotoxic effects of DHA+CQ, LNP/DC, and RLNP/DC in HCT116 cells *in vitro*, RLNP/DC exhibited the most significant inhibitory effect on primary colorectal tumor growth *in vivo*.

In addition, the inhibitory effect of DHA and CQ combination therapy on liver metastasis was further investigated in the mouse model. DHA and CQ combination therapy and LNPs considerably attenuated liver metastasis, as indicated by the metastatic nodes. Consistent with the previous reports on anti-metastasis effect of DHA and CQ, we proposed that the synergistic effects of DHA and CQ combination were mainly attributable to prevention of tumor angiogenesis and cell detachment from the primary tumor. In addition, enhanced cytotoxic effect of the drug combination on tumor cells contributed to reduced liver metastasis. The present study demonstrated that the synergistic anti-metastasis effect on colorectal cancer was mediated by downregulation of VEGF by both DHA and CQ and upregulation of paxillin to inhibit tumor angiogenesis and maintain focal adhesion. Intriguingly, RLNP markedly improved anti-metastasis effect of DHA and CQ both *in vitro* and *in vivo*, which was verified with no anti-tumor and anti-metastasis effects alone in the colorectal cancer model. It is reasonable to conclude that the RLNPs developed in this study have great potential to overcome various physiological barriers and reach the orthotopic and metastatic tumor lesions, therefore, RLNPs serve as an ideal drug delivery system for DHA and CQ combination to exert optimal therapeutic effects against colorectal cancer. Clinical application of RLNP/DC requires further optimization of dosage and administration intervals and evaluation of long-term toxicity.

**FIGURE 8:**
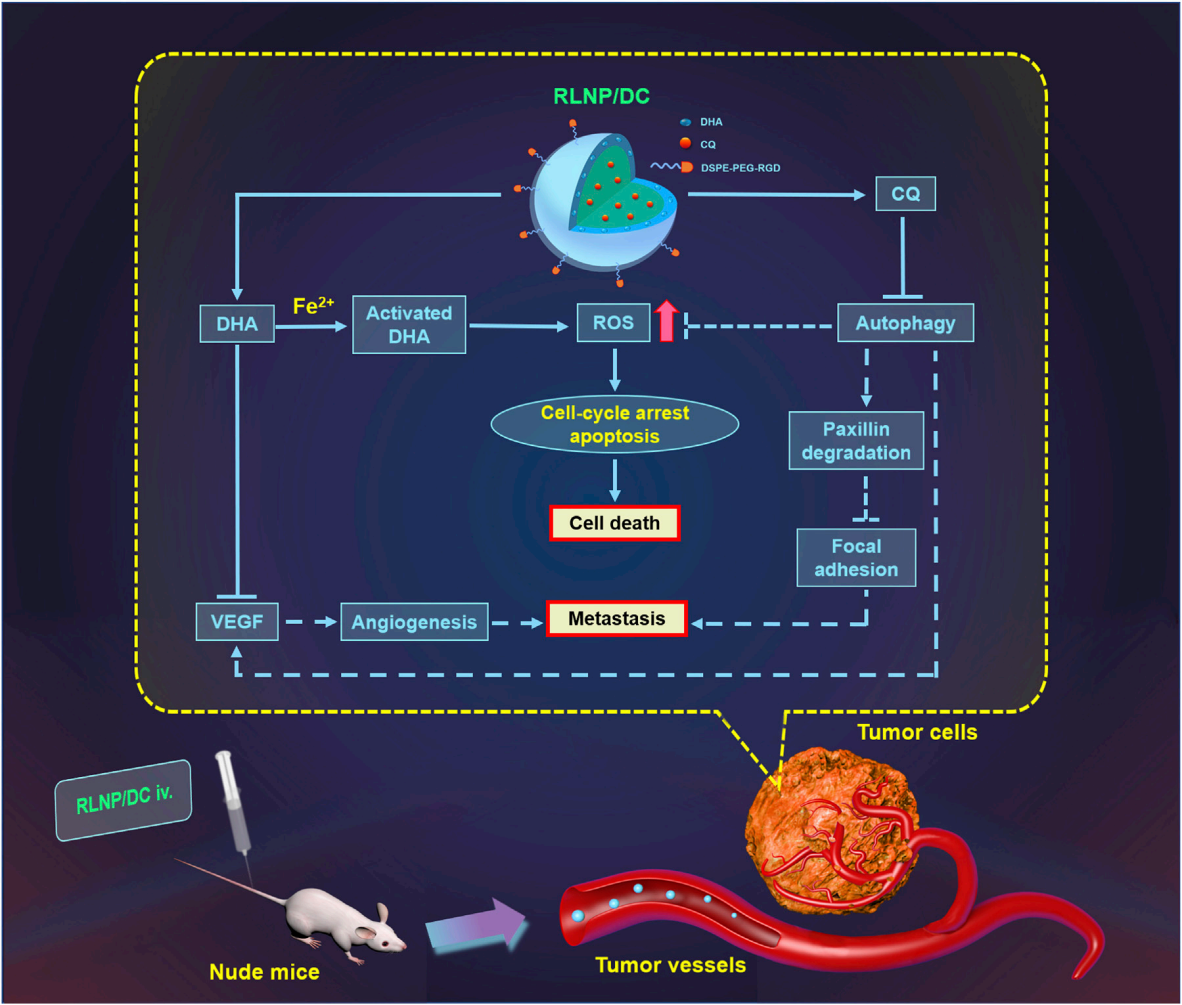
Schematic illustration of suppression of proliferation and liver metastasis of colorectal cancer by RLNP/DC. DHA is activated by Fe^2+^ to generate ROS and CQ blocked ROS elimination via autophagy inhibition, which contributed to irreversible upregulation of intracellular ROS level leading to cell death. Moreover, both DHA and CQ exerted anti-angiogenesis activity via down regulation of VEGF, and CQ prevented the degradation of paxillin leading to enhanced focal adhesion of tumor cells to the ECM, resulting in anti-metastasis effect on colorectal cells. RLNP plays an important role for targeted co-delivery of DHA and CQ to the primary tumor region aiming at improved anti-tumor efficacy against colorectal cancer.

In summary, we have developed RLNPs, a dual-sensitive nanocarrier, for the targeted delivery of DHA and CQ to suppress the proliferation and liver metastasis of colorectal cancer synergistically (Figure 8). DHA and CQ enhanced intracellular ROS levels, which not only led to cell death but also further promoted the drug release owing to the ROS-sensitivity of LNPs. Moreover, DHA and CQ inhibited angiogenesis and enhanced focal adhesion, resulting in marked suppression of liver metastasis. Thus, DHA and CQ combination therapy holds promise for the treatment of colorectal cancer in the clinical setting by virtue of RLNP nanocarriers.

## Data Availability

The original contributions presented in the study are included in the article/Supplementary Material, further inquiries can be directed to the corresponding authors.
