# Supplementary material for: Targeted Lipid Nanoparticles Encapsulating Dihydroartemisinin and Chloroquine Phosphate for Suppressing the Proliferation and Liver Metastasis of Colorectal Cancer
Source: Front Pharmacol. 2021 Oct 8;12:720777. doi: 10.3389/fphar.2021.720777 (PMC8531263; doi:10.3389/fphar.2021.720777)
Supplement: Supplementary file 1 [file DataSheet1.DOCX]

Supplementary Material

**Targeted lipid nanoparticles encapsulating dihydroartemisinin and chloroquine phosphate for suppressing the proliferation and liver metastasis of colorectal cancer**

Supplementary Figures

**
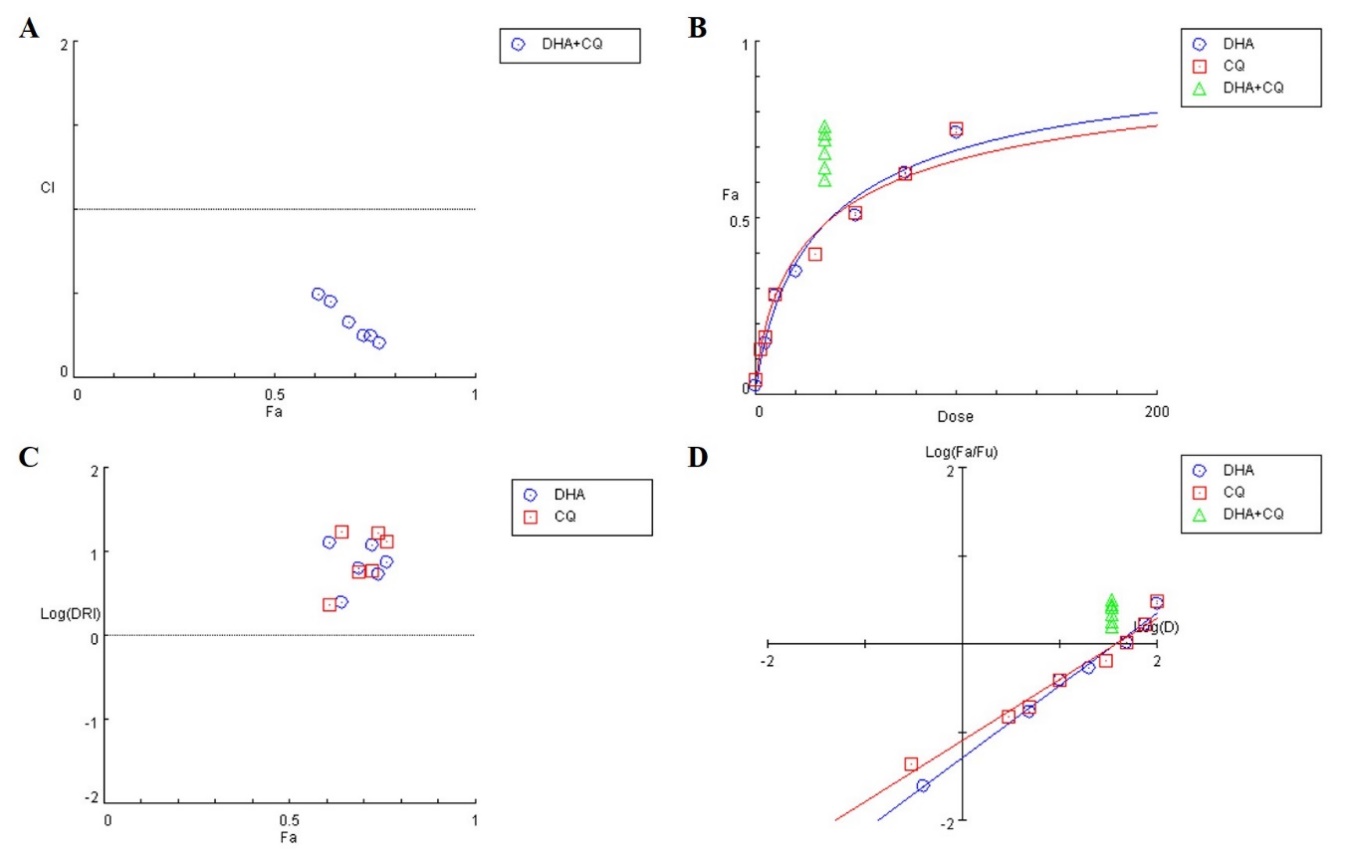
**

**Supplementary Figure 1.** CompuSyn analysis of the cytotoxicity of DHA and CQ on HCT116 cells. (A) The CI plot of DHA and CQ combination. (B) The dose-effect curve of DHA and CQ combination. (C) Dose reduction index (DRI) plot for DHA and CQ combination. (D) Median-effect plot for DHA and CQ combination.


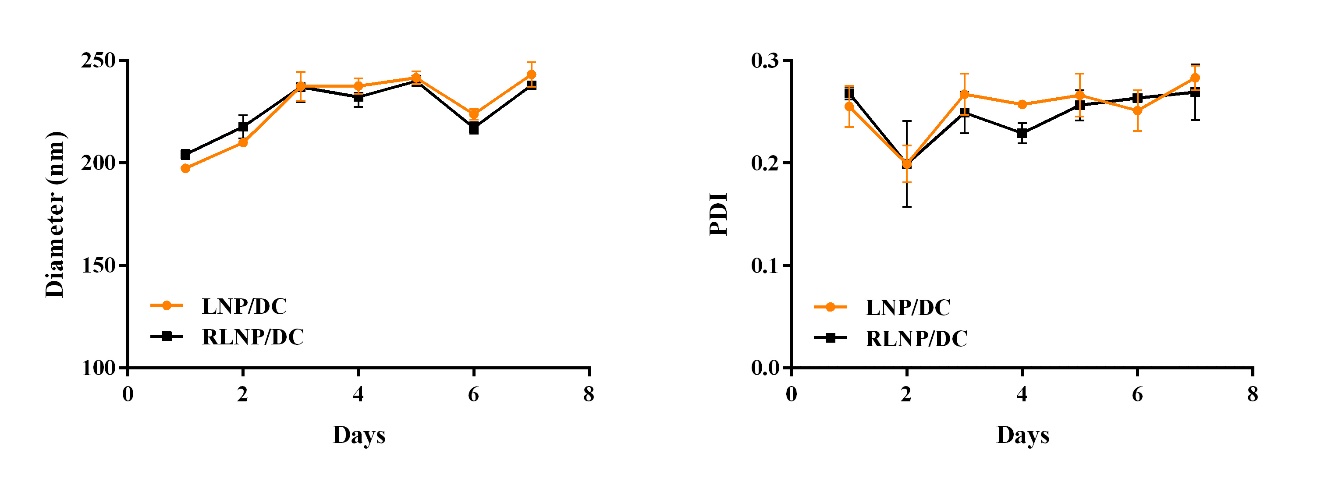


**Supplementary Figure 2.** Detection on diameter (**A**) and PDI (**B**) of LNP/DC and RLNP/DC in 4 ℃ for 7 days.


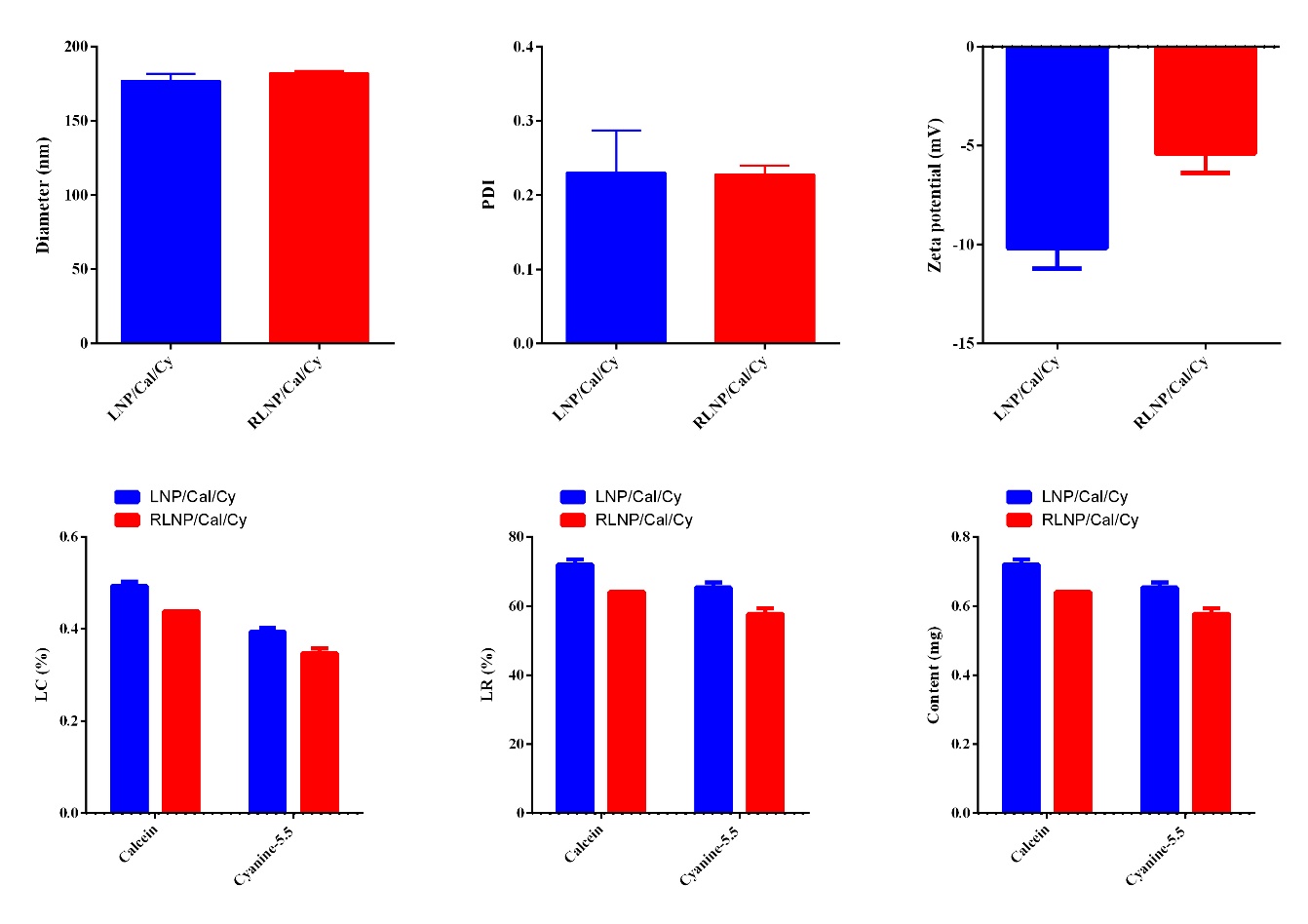


**Supplementary Figure 3.** Characterization of LNP/Cal/Cy and RLNP/Cal/Cy. Diameter (**A**), PDI (**B**), zeta potential (**C**), LC (**D**), LR (**E**) and content (**F**) of calcein/cyanine-5.5.


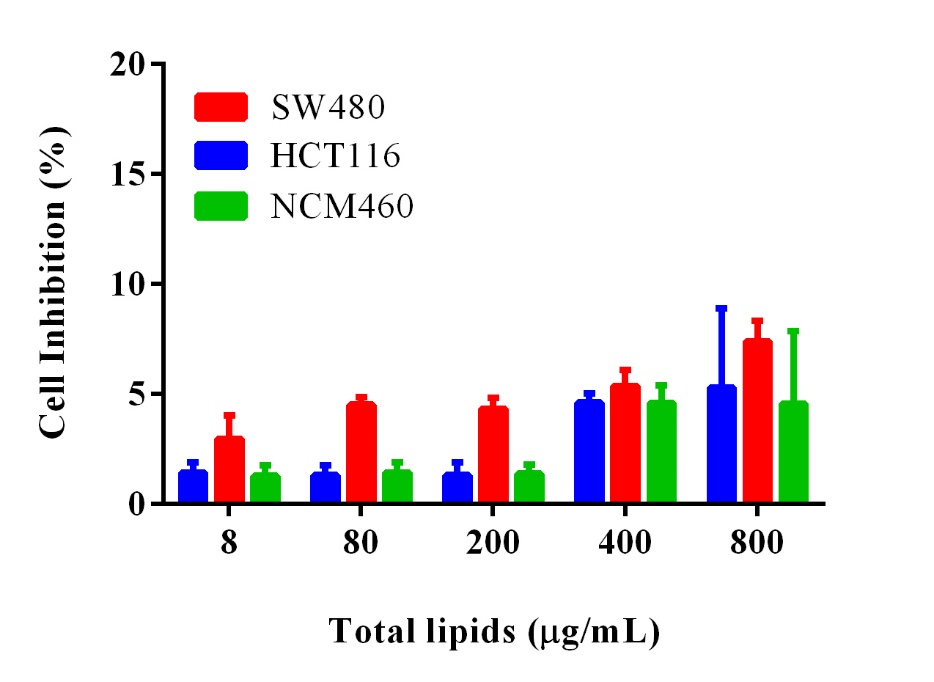


**Supplementary Figure 4.** Cytotoxicity of RLNP on HCT116, SW480 and NCM460 cells at different phospholipid concentrations. Each bar was shown as means ± SD (*n* = 5).
